# Supplementary material for: Thermodynamically inconsistent extreme precipitation sensitivities across continents driven by cloud-radiative effects
Source: Nat Commun. 2024 Dec 11;15:10669. doi: 10.1038/s41467-024-55143-8 (PMC11634894; doi:10.1038/s41467-024-55143-8)
Supplement: Supplementary file 1 — Supplementary Information [file 41467_2024_55143_MOESM1_ESM.pdf]

Supporting Information for

Thermodynamically inconsistent extreme precipitation sensitivities across continents driven by cloud-radiative effects

Sarosh Alam Ghausi[1,2,3], Erwin Zehe[3], Subimal Ghosh[4,5], Yinglin Tian[6] and Axel Kleidon[1]

[1] Biospheric Theory and Modelling Group, Max Planck Institute for Biogeochemistry, Jena 07745, Germany.

[2] International Max Planck Research School for Global Biogeochemical Cycles (IMPRS – gBGC), Jena 07745, Germany.

[3] Institute of Water Resources and River Basin Management, Karlsruhe Institute of Technology – KIT, Karlsruhe, Germany

[4] Department of Civil Engineering, Indian Institute of Technology Bombay.

[5] Interdisciplinary Programme in Climate Studies, Indian Institute of Technology Bombay.

[6] Department of Earth System Analysis, Potsdam Institute for Climate Impact Research (PIK) – Member of the Leibniz Association, Potsdam, 14473, Germany

Corresponding author: Sarosh Alam Ghausi (sghausi@bgc-jena.mpg.de)

This PDF file includes:

Figures from S1 to S16

Figure: S1

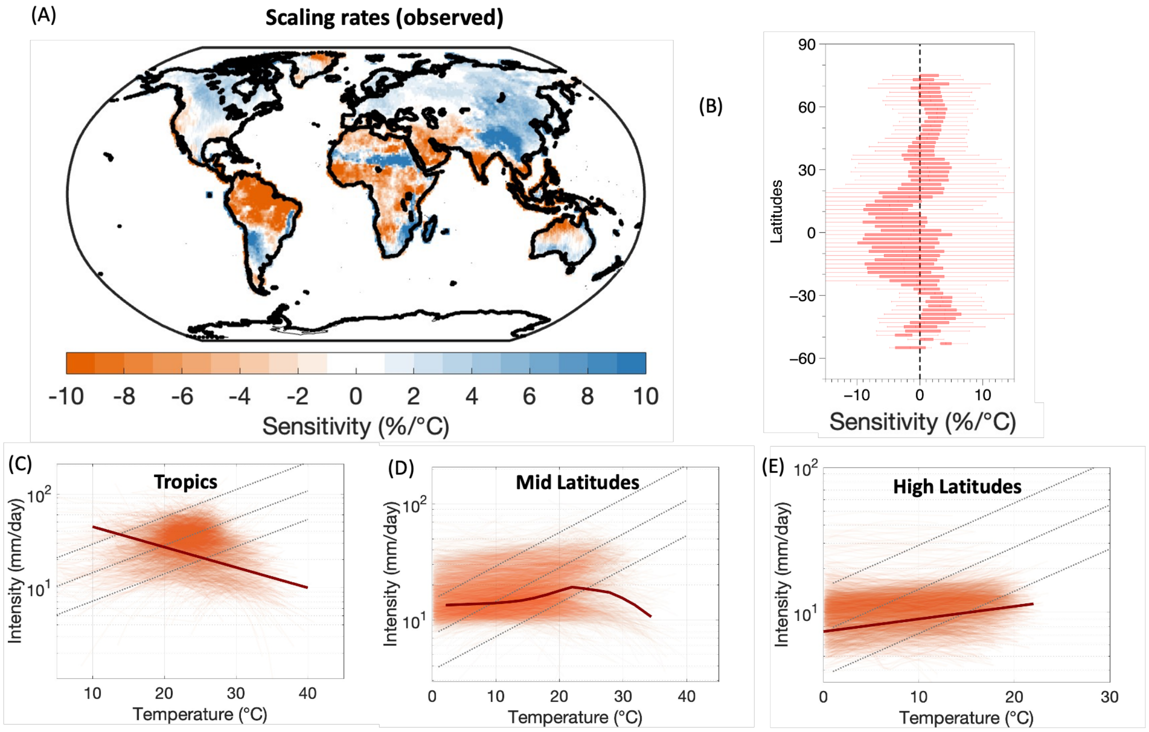

Figure S1: same as figure 1 but with GPCP rainfall data.

Figure: S2

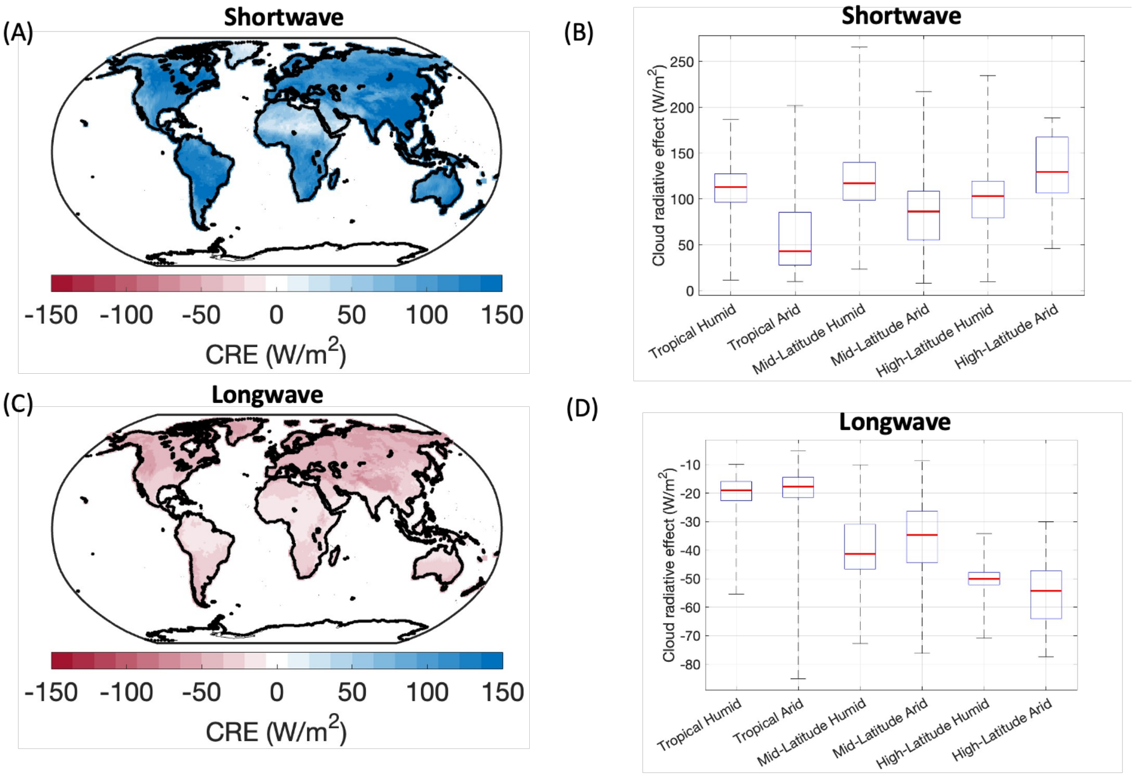

Figure S2: Global distribution of cloud radiative effects (defined as the difference between the "clear-sky" and "all-sky" radiative fluxes) for absorbed shortwave (A) and downwelling longwave (C) radiation. Box plots for shortwave CRE (B) and downwelling longwave CRE (D) separated into different zones. The tropical regions are classified as grids between 23S and 23N, mid-latitudes as grids from 23S-55S and 23N-55N and high-latitudes as grids beyond 55S and 55N. The classification into humid and arid regions is derived from the Budyko Aridity index, which is calculated as the ratio of mean annual potential evapotranspiration to mean annual precipitation. The region is classified as humid when aridity index is less than 1 and arid when it is greater than 1.

Figure: S3

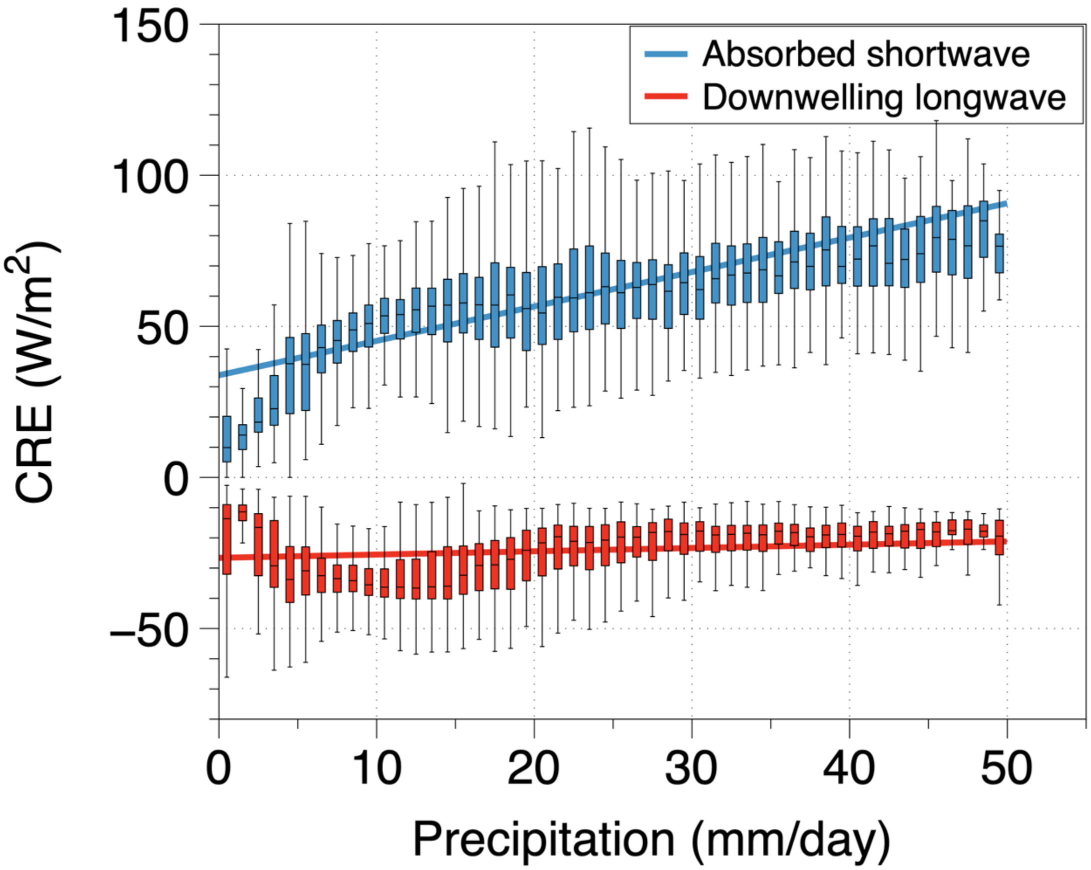

Figure S3: Variation of cloud radiative effects with precipitation for absorbed shortwave (blue) and Downwelling longwave (red) radiation.

Figure S4

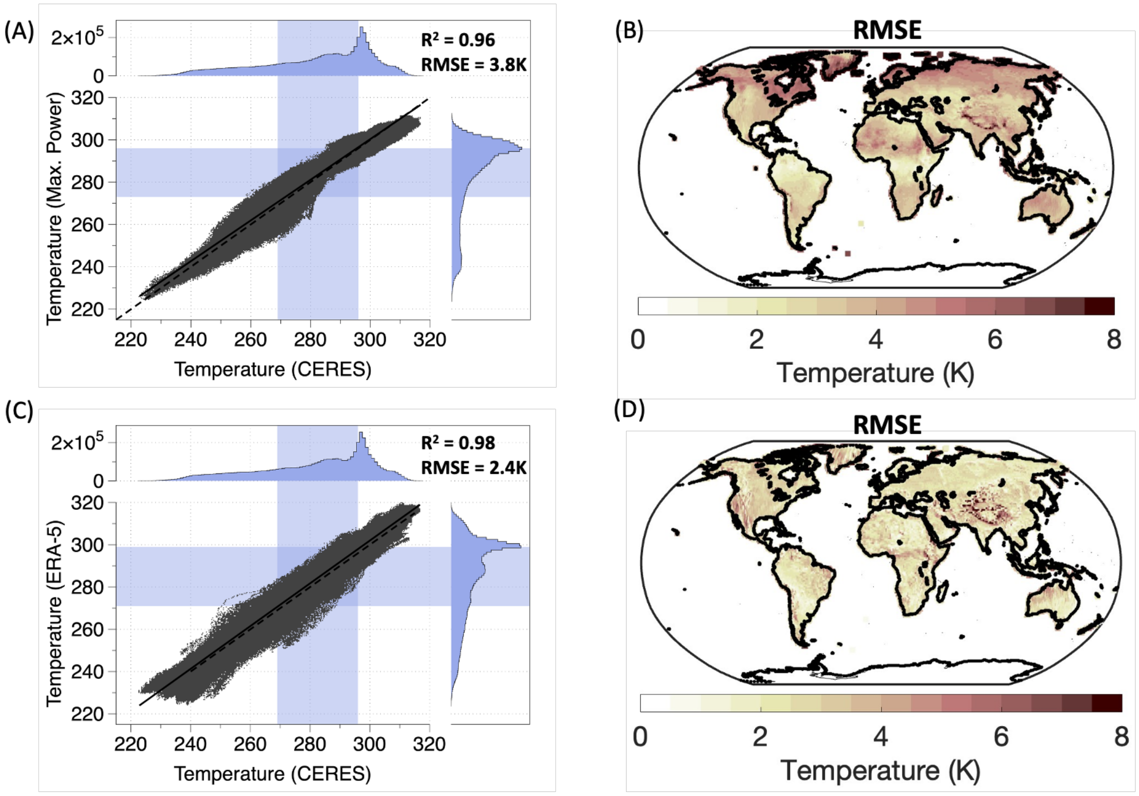

Figure S4: (A) Comparison of daily estimated surface temperatures using the thermodynamically constrained surface energy balance model against observations from the NASA-CERES dataset. (B) Global map of root mean squared error (RMSE) between the estimated surface temperatures and NASA-CERES observations, computed for the daily timeseries at each grid point. (C, D) same as (A, B) but for the comparison between ERA-5 reanalysis data and NASA-CERES observations. Note: The surface temperature was diagnosed from the upwelling longwave radiation from NASA-CERES dataset using the Stefan-Boltzmann law.

Figure S5

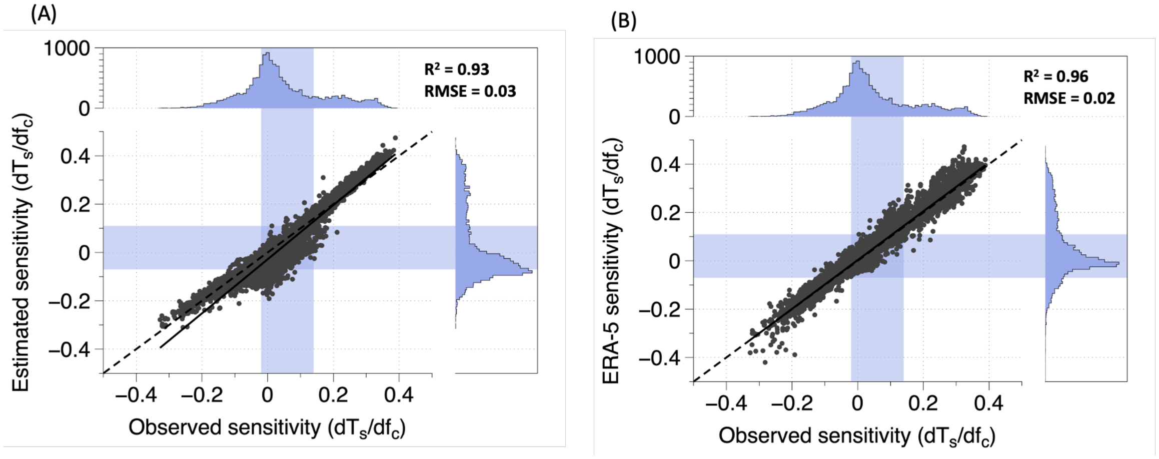

Figure S5: (A) Comparison of the first-order sensitivity of daily surface temperatures to changes in cloud cover, estimated by the thermodynamically constrained energy balance model, with those derived from NASA-CERES observations. Each black dot represents the sensitivity at each grid point over land. (B) Same as (A) but for the comparison of cloud-cover sensitivities of ERA-5 surface temperatures with those derived from CERES observations.

Figure: S6

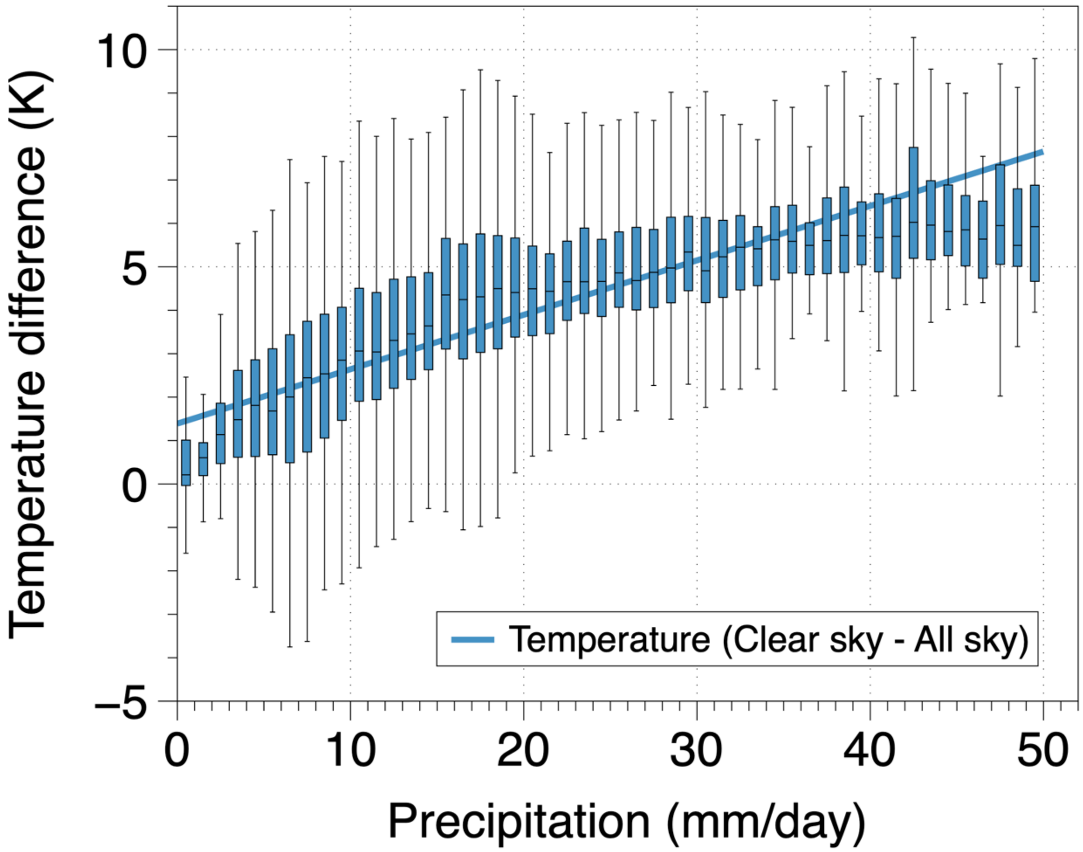

Figure S6: Variation of estimated temperature difference between "clear-sky" and "all-sky" conditions (K) as a function of precipitation. Note: positive temperature difference indicates cooling.

Figure S7

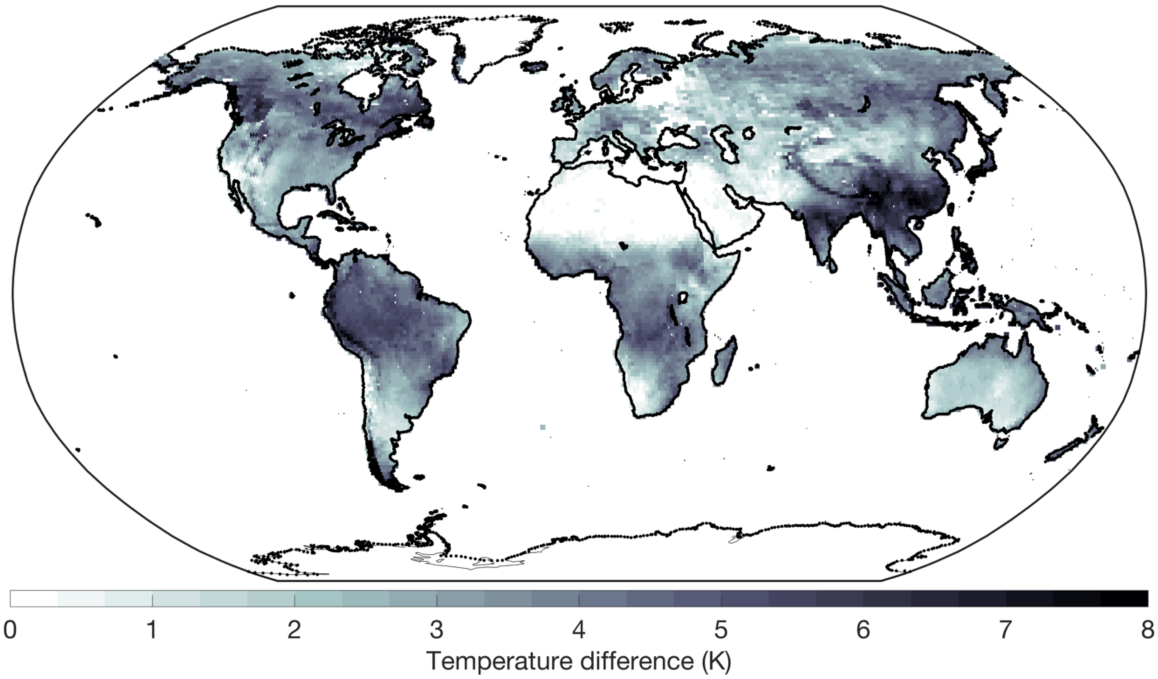

Figure S7: Global map of temperature difference between "clear-sky" and "all-sky" conditions.

Figure: S8

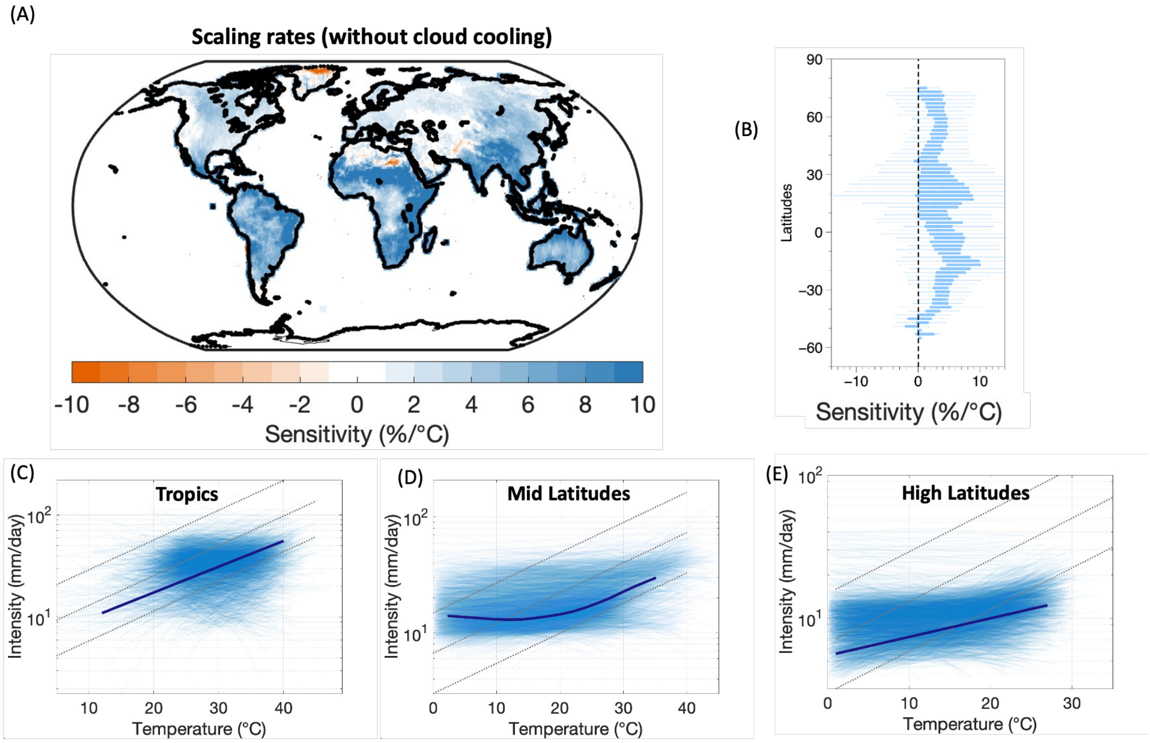

Figure S8: same as figure 3 but with GPCP rainfall data.

Figure S9

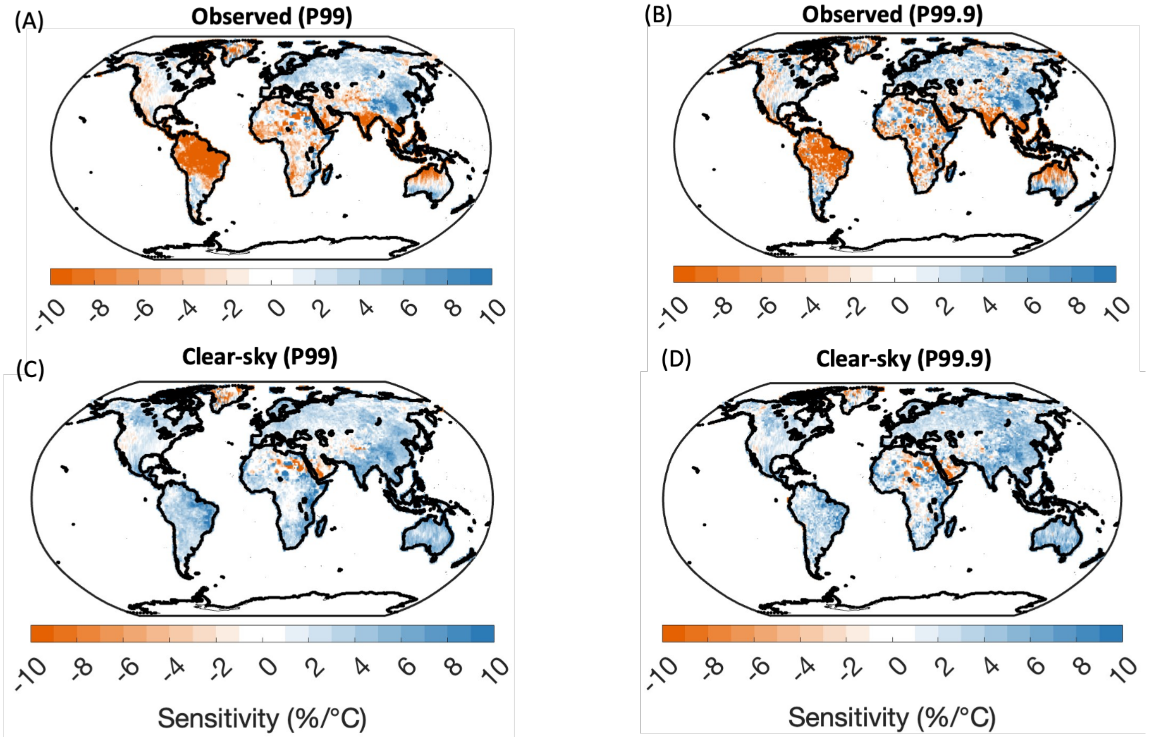

Figure S9: Global map of extreme precipitation – temperature (EP-T) sensitivities for 99th percentile rainfall (A, C) and 99.9th percentile rainfall (B, D) with observed temperatures (A, B) and with corrected temperatures for cloud-effects (C, D). Note the rainfall and temperature data is from CPC.

Figure S10

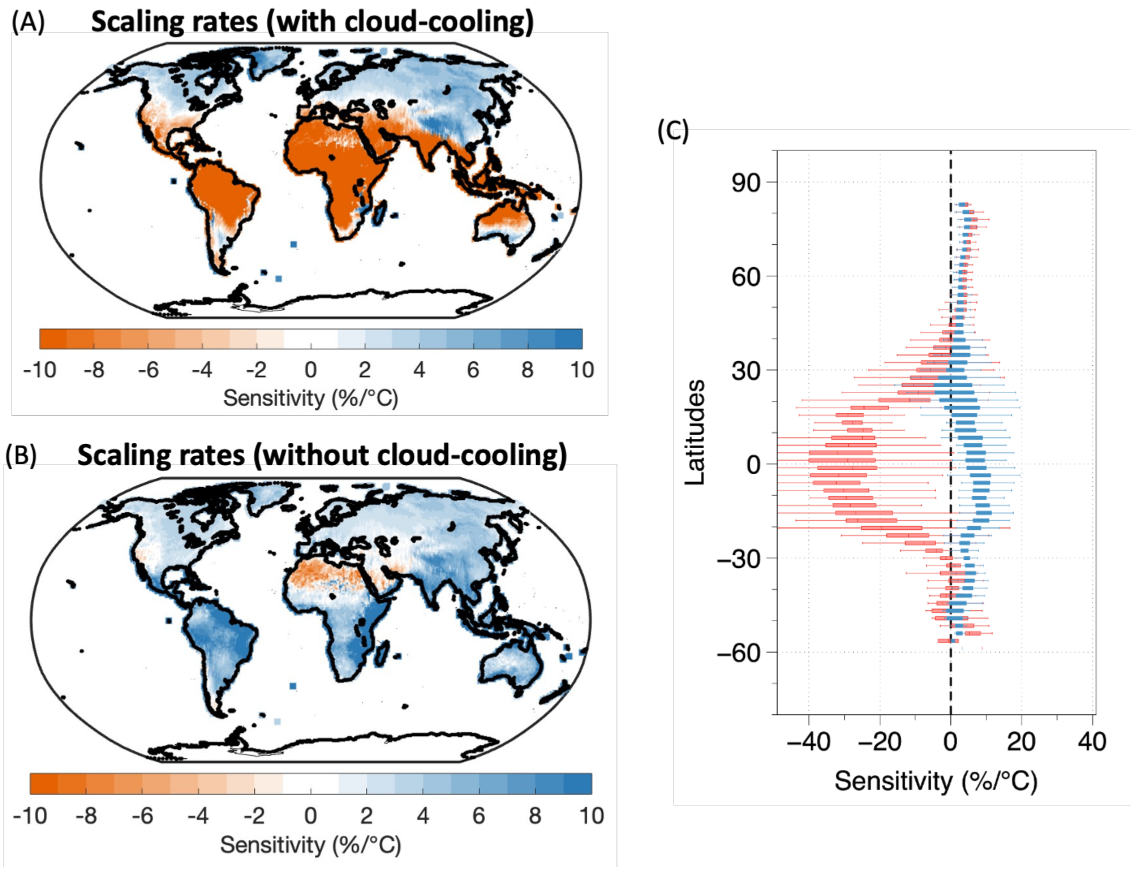

Figure S10: Global map of extreme precipitation (P95) – temperature (EP-T) sensitivities estimated from ERA-5 data using temperatures (A) with cloud-cooling and (B) after correcting for cloud-cooling, (C) zonal variation of estimated EP-T sensitivities with and without the cloud effects as red and blue respectively.

Figure S11

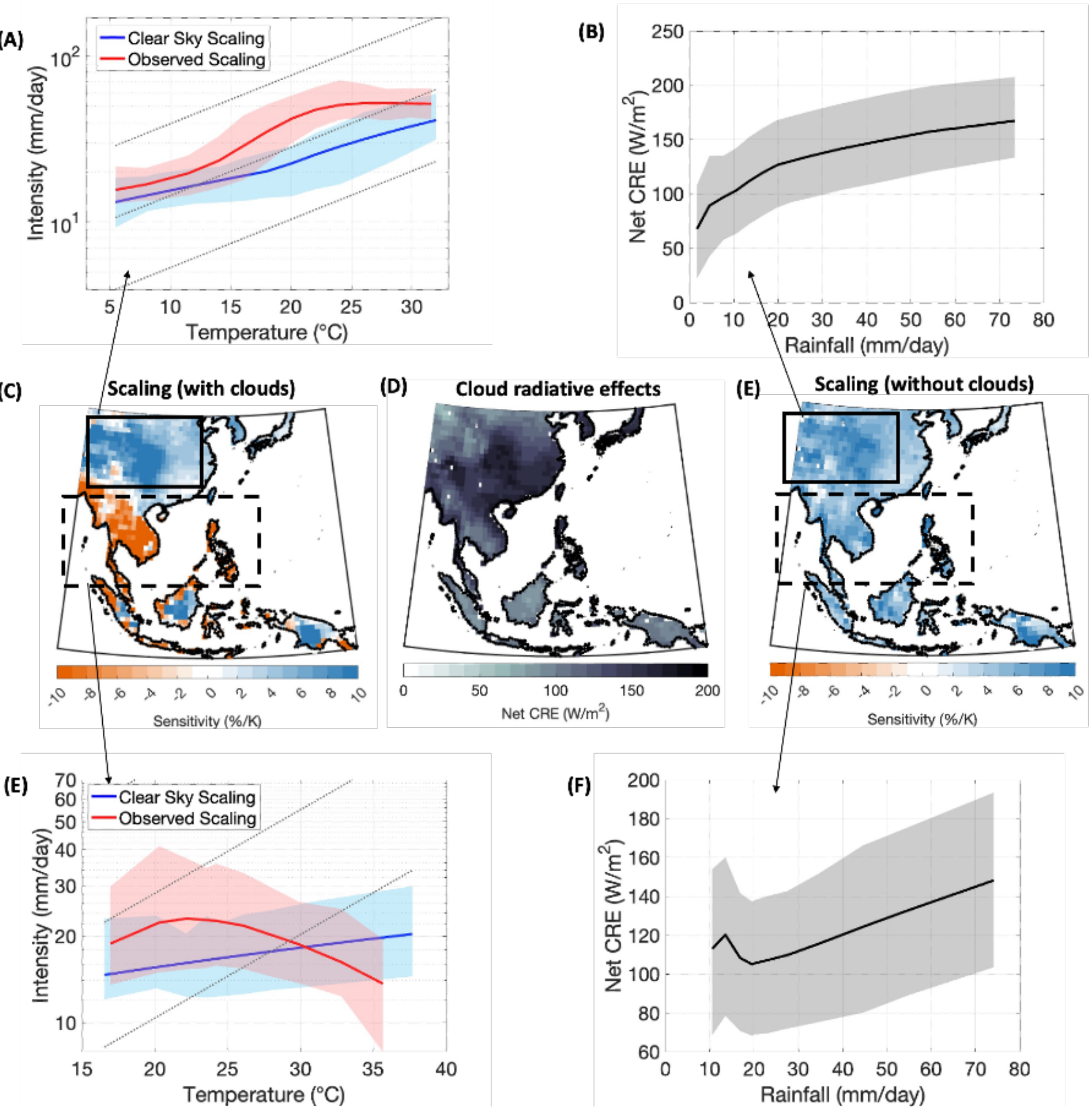

Figure S11: Maps of EP-T sensitivities for observations (C), Net cloud radiative effect on extreme rainfall (P95) days (D) and EP-T sensitivities after correcting for cloud-effects (E) isolated over Southeast Asia region. Black solid and black dotted boxes further separate these regions into two parts. (A, E) shows the scaling curves of extreme precipitation with observed temperatures (red line) and temperatures corrected for cloud-effects (blue-line) for grids within black solid box and black dotted box respectively. (B, F) shows the variation of net cloud radiative effect with rainfall for grids within black solid box and black dotted box respectively.

Figure S12

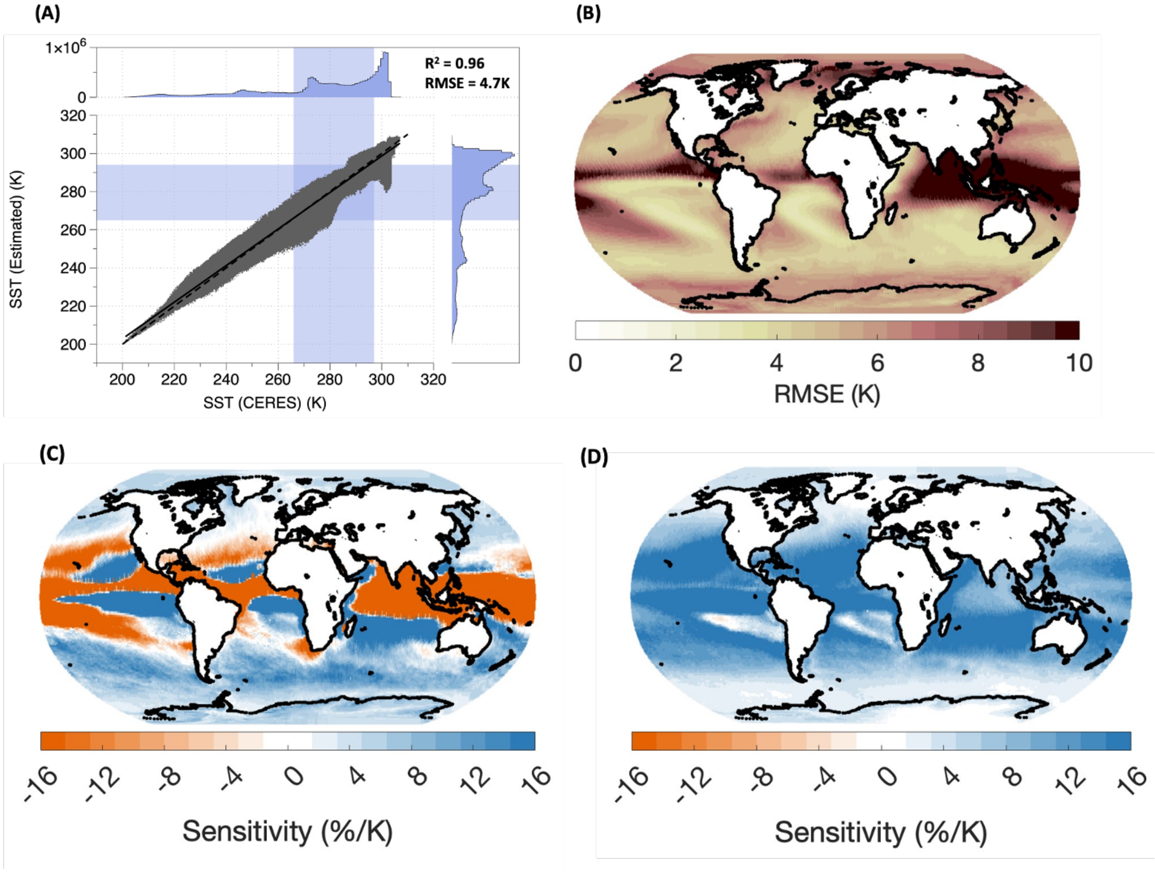

Figure S12: (A) Comparison of estimated daily sea surface temperatures with the energy balance model with temperatures derived from (NASA-CERES). Root mean squared error (RMSE) between daily estimated and observed temperatures from CERES across all the oceanic grids. Global map of extreme precipitation-temperature sensitivities over oceanic grids with (C) observed temperatures and (D) temperatures corrected for cloud-effects.

Figure S13

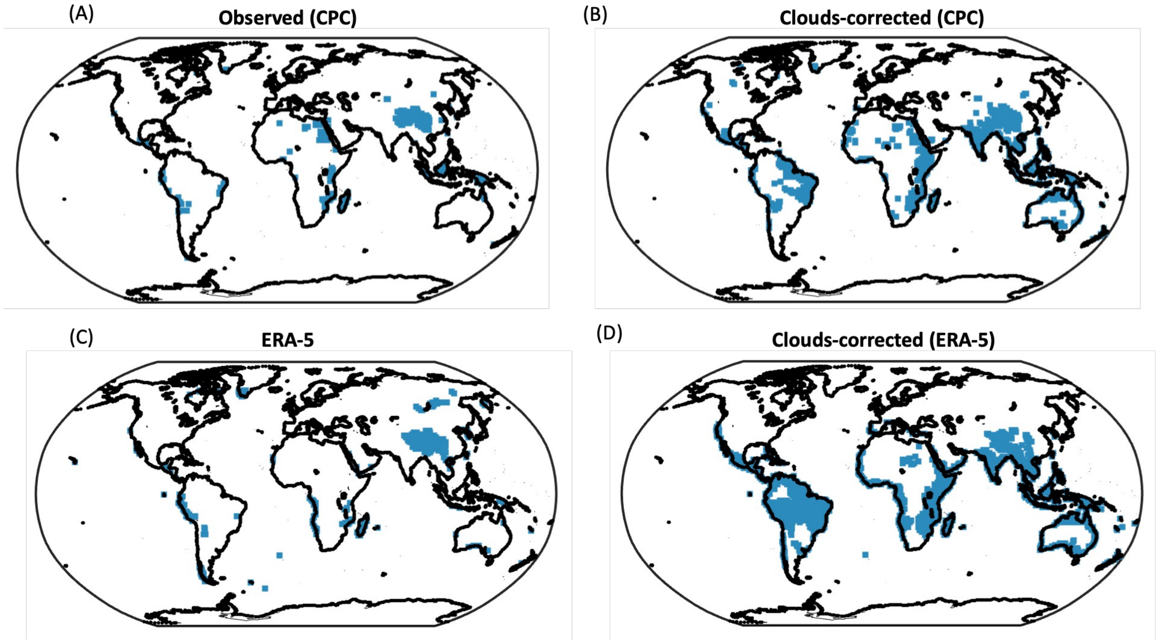

Figure S13: Grids exhibiting super CC scaling (EP-T sensitivity > 7%/°C) in observations (A, B) and ERA-5 reanalysis (C, D) with cloud-effects (A, C) and after correcting for cloud-effects (B, D).

Figure S14

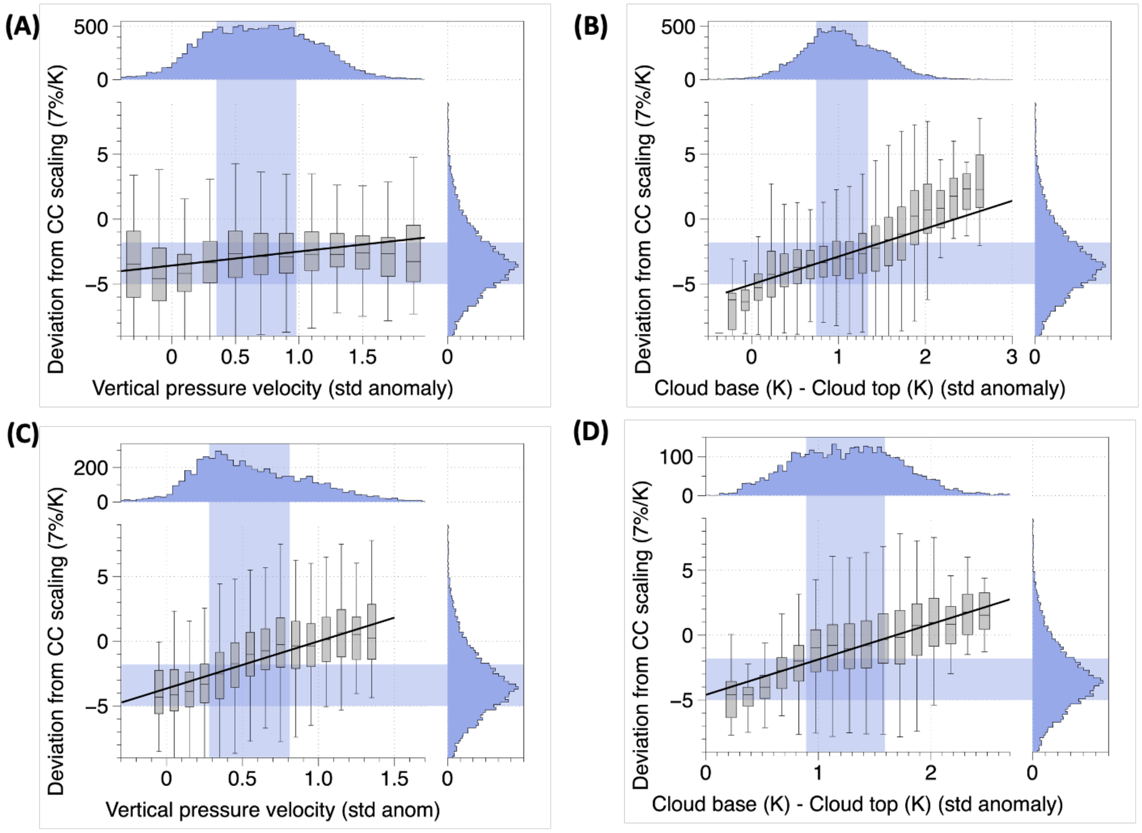

Figure S14: Deviation in EP-T sensitivities (P95) from CC rate (7%/°C) after correcting for cloud-effects as a function of (A) standardized anomalies in vertical pressure velocity at 650 hpa on extreme rainfall (P95) days and (B) standardized anomalies in the temperature difference between cloud-base and cloud-top on extreme rainfall days (P95). (C, D) same as (A, B) but for grids between 30S and 30N.

Figure S15

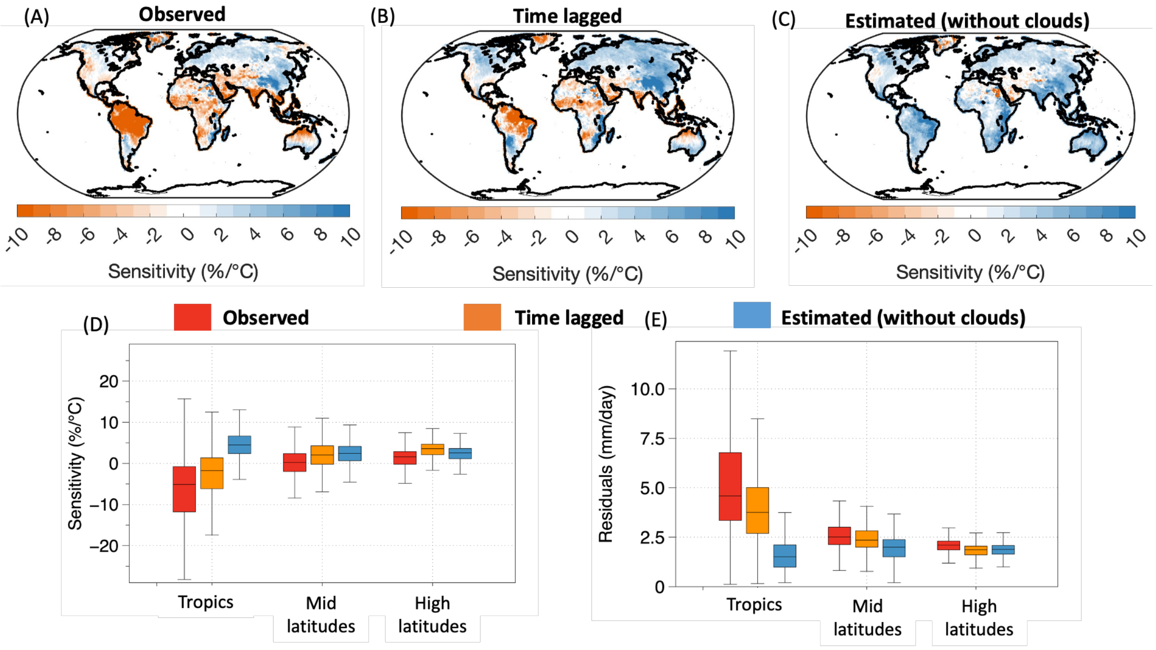

Figure S15: (Top row) Global map of extreme precipitation-temperature sensitivities estimated using quantile regression with (A) observed temperatures, (B) 1-day time lagged temperatures and (C) temperatures corrected for cloud-radiative effects. (Bottom row-D) Comparison of EP-T scaling rates estimated using observed temperatures (red), time-lagged temperatures (orange) and with temperatures corrected for the cloud-cooling effect (blue) for tropics, mid-latitudes and high-latitudes. (E) same as (D) but for the residuals between observations and fitted quantile regression

Figure S16

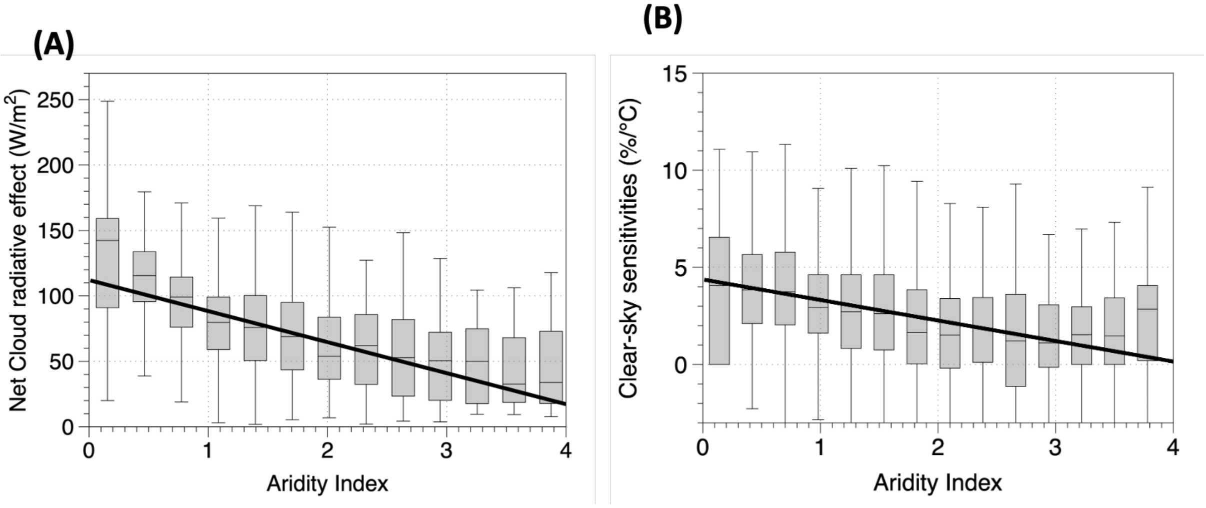

Figure S16: Variation along the Budyko aridity index (defined as the ratio between mean net radiation and the energy equivalent to mean annual precipitation, where higher values (> 1) indicate more arid conditions) of (A) Net Cloud radiative effects (W/m^2) and (B) Extreme precipitation-temperature scaling after removing the effect of clouds.
